# Atherosclerosis T1-weighted characterization (CATCH): evaluation of the accuracy for identifying intraplaque hemorrhage with histological validation in carotid and coronary artery specimens

**DOI:** 10.1186/s12968-018-0447-x

**Published:** 2018-04-26

**Authors:** Wen Liu, Yibin Xie, Chuan Wang, Yanni Du, Christopher Nguyen, Zhenjia Wang, Zhaoyang Fan, Li Dong, Yi Liu, Xiaoming Bi, Jing An, Chengxiong Gu, Wei Yu, Debiao Li

**Affiliations:** 1 Department of Radiology, Beijing Anzhen Hospital, Capital Medical University, Beijing, China; 2 Biomedical Imaging Research Institute, Cedars-Sinai Medical Center, Los Angeles, CA USA; 3 Department of Cardiac Surgery, Anzhen Hospital, Capital Medical University, Beijing, China; 4 Departments of Medicine and Bioengineering, University of California, Los Angeles, CA USA; 5 MR R&D, Siemens Healthineers, Los Angeles, CA USA; 6 MR Collaborations NE Asia, Siemens Healthcare, Beijing, China; 7 Cardiovascular Research Center, Massachusetts General Hospital, Harvard Medical School, Charlestown, MA USA

**Keywords:** Coronary high intensity plaques (CHIPs), T1w imaging, Intraplaque hemorrhage, CATCH

## Abstract

**Background:**

Coronary high intensity plaques (CHIPs) detected using cardiovascular magnetic resonance (CMR) coronary atherosclerosis T1-weighted characterization with integrated anatomical reference (CATCH) have been shown to be positively associated with high-risk morphology observed on intracoronary optical coherence tomography (OCT). This study sought to validate whether CHIPs detected on CATCH indicate the presence of intraplaque hemorrhage (IPH) through ex vivo imaging of carotid and coronary plaque specimens, with histopathology as the standard reference.

**Methods:**

Ten patients scheduled to undergo carotid endarterectomy underwent CMR with the conventional T1-weighted (T1w) sequence. Eleven carotid atherosclerotic plaques removed at carotid endarterectomy and six coronary artery endarterectomy specimens removed from patients undergoing coronary artery bypass grafting (CABG) were scanned ex vivo using both the conventional T1w sequence and CATCH. Both in vivo and ex vivo images were examined for the presence of IPH. The sensitivity, specificity, and Cohen Kappa (k) value of each scan were calculated using matched histological sections as the reference. k value between each scan in the discrimination of IPH was also computed.

**Results:**

A total of 236 in vivo locations, 328 ex vivo and matching histology locations were included for the analysis. Sensitivity, specificity, and k value were 76.7%, 95.3%, and 0.75 for in vivo T1w imaging, 77.2%, 97.4%, and 0.78 for ex vivo T1w imaging, and 95.0%, 92.1%, and 0.84 for ex vivo CATCH, respectively. Moderate agreement was reached between in vivo T1w imaging, ex vivo T1w imaging, and ex vivo CATCH for the detection of IPH: between in vivo T1w imaging and ex vivo CATCH (k = 0.68), between ex vivo T1w imaging and ex vivo CATCH (k = 0.74), between in vivo T1w imaging and ex vivo T1w imaging (k = 0.83). None of the coronary artery plaque locations showed IPH.

**Conclusion:**

This study demonstrated that carotid CHIPs detected by CATCH can be used to assess for IPH, a high-risk plaque feature.

## Background

Acute coronary syndrome (ACS) arises from an indolent cardiovascular disease progression process and accounts for > 50% of sudden cardiac deaths [1]. Rupture or erosion of the endothelial surface of an atherosclerotic plaque with subsequent thrombosis is believed to be the most common mechanism in ACS [2]. A noninvasive and non-ionizing radiation imaging tool that enables the detection of coronary plaques prone to disruption is highly desirable for risk stratification and for evaluating the response to therapeutic interventions.

During the past decade, non-contrast T1-weighted (T1w) cardiovascular magnetic resonance (CMR) imaging with inversion-recovery (IR) sequence for plaque imaging has emerged as a novel non-invasive imaging technique for the identification of high-risk coronary plaques. Previous studies have shown that high intensity plaques in coronary artery on T1w CMR images are associated with future cardiac events [3, 4]. In spite of the prospective prognostic ability for conventional T1w images to evaluate coronary atherosclerosis, there are a few technical barriers preventing clinical translation, such as lack of anatomical reference, low spatial resolution, and long, unpredictable scan time. Recently, a novel three dimensional (3D) whole-heart imaging technique, coronary atherosclerosis T1-weighted characterization with integrated anatomical reference (CATCH) was developed to address the aforementioned limitations for coronary atherosclerotic plaque characterization [5]. It used IR-prepared spoiled gradient echo acquisition and could simultaneously obtain T1w images along with reference bright-blood coronary CMR angiogram with 100% respiratory gating efficiency. Comparison with optical coherence tomography in patients indicated the positive association between coronary high intensity plaques (CHIPs) on CATCH and vulnerable plaque features such as lipid core and macrophage accumulation. Based on previous observations in carotid plaque imaging, CHIPs on T1w images may indicate the presence of intraplaque hemorrhage (IPH), another important biomarker for high risk plaques [6]. However, due to the lack of gold standard for detecting IPH in vivo in the coronary arteries, the potential link between CHIPs and coronary IPH has not been established. The purpose of this work was to investigate the association between CHIPs on CATCH and the presence of IPH through ex vivo imaging of carotid and coronary plaque specimens.

## Methods

### Study population and sample procurement

Between April 2016 and September 2017, 12 patients scheduled to undergo carotid endarterectomy and 7 patients underwent coronary artery endarterectomy with coronary artery bypass grafting (CABG) (61.6 ± 8.6 years) from the departments of vascular surgery and cardiac surgery, respectively, were prospectively recruited with informed consent. Eleven patients scheduled to undergo carotid endarterectomy received carotid artery CMR imaging within 5 days before the surgical procedure. One patient failed to undergo CMR examination before carotid endarterectomy.

After carotid endarterectomy or coronary artery endarterectomy, 12 carotid and 7 coronary atherosclerotic plaques were obtained. To preserve excised plaques, samples were placed in 0.9% saline solution immediately after being removed from patients. To preserve the relaxation parameters of the in vivo tissue while not interrupting the histological processing, the ex vivo CMR study was conducted within 3 h after surgery. After ex vivo CMR imaging, the samples were immediately fixed in formalin.

**Fig. 1:**
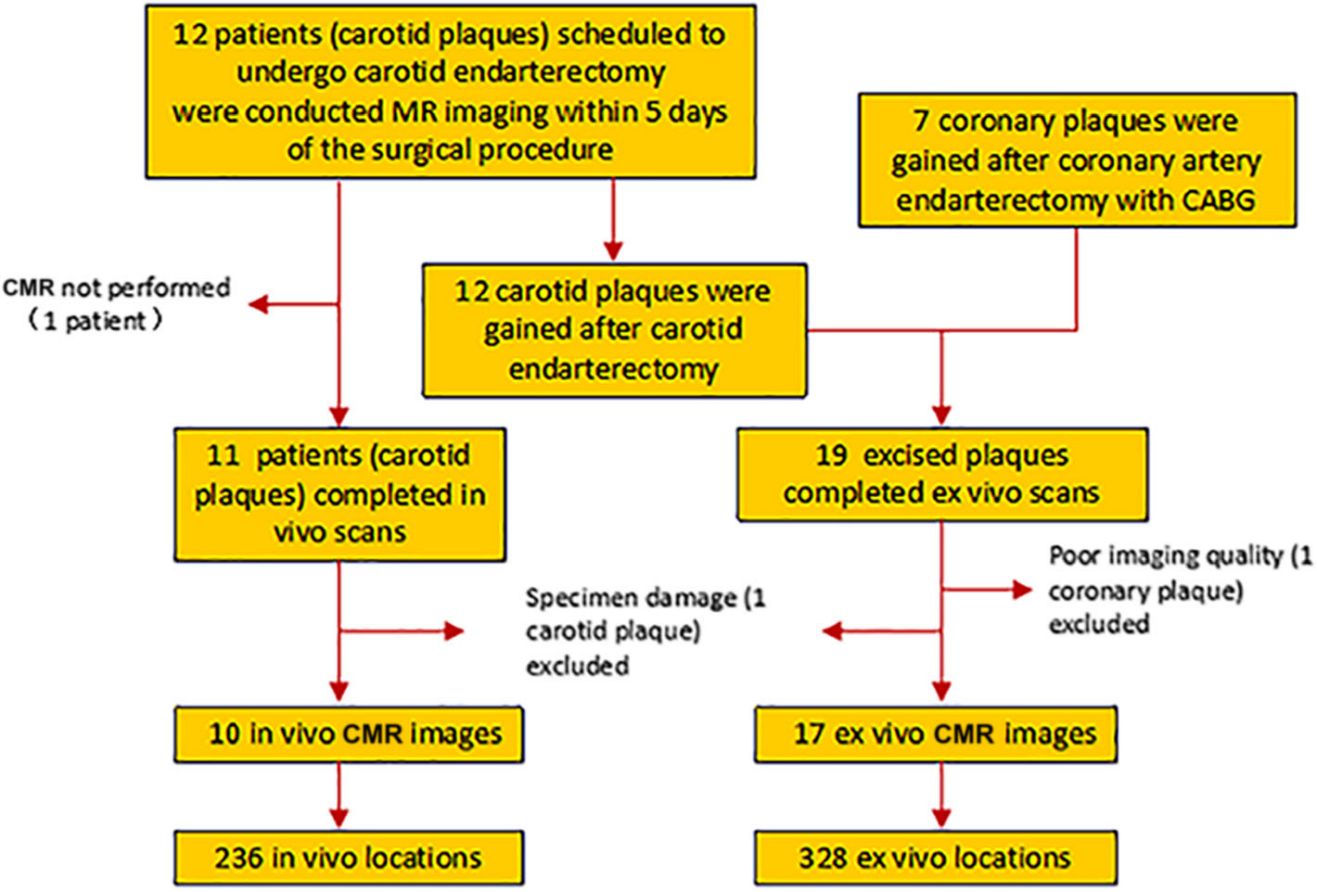
Flow Chart of Inclusion Criteria. Twelve patients scheduled to undergo carotid endarterectomy and 7 patients underwent coronary artery endarterectomy with coronary artery bypass grafting (CABG) were recruited for the study, while11 patients scheduled to undergo carotid endarterectomy successfully completed T1w images and T2w images in vivo scans. After carotid endarterectomy or coronary artery endarterectomy with CABG, 12 carotid and 7 coronary atherosclerotic plaques respectively were obtained for ex vivo T1w images, ex vivo T2w images and ex vivo CATCH scans. Among the 19 subjects enrolled initially, 2 were excluded from the ultimate analysis: one (coronary plaque) was due to poor imaging quality, while the other (carotid plaque) failed to histology matching owing to specimen damage. Ultimately, 10 in vivo plaques and 17 ex vivo plaques were included for analysis, yielding a total of 236 in vivo locations, 328 ex vivo and matched histology locations

Among the 19 subjects enrolled initially, 2 were excluded from the final analysis: one (coronary plaque) due to poor ex vivo image quality because of delayed preservation of excised plaque resulting in severe tissue distortion; and one carotid plaque due to the failure to match histology because of specimen damage (Fig. 1).

### CMR imaging protocols

For in vivo imaging, the CMR scanning was performed on a 3 Tesla (MAGNETOM Verio, Siemens Healthineers, Erlangen, Germany) using an 8-channel phased-array carotid surface coil (Shanghai Chenguang Medical technologies CO, LTD, Shanghai, China). Axial T1w images and T2 weighted (T2w) images centered around the carotid bifurcation were acquired for a total longitudinal coverage of 24 mm. The parameters of turbo spin echo T1w images were as follows: TE/TR = 9.4/720 ms, in-plane resolution = 0.6 × 0.6 mm^2^, number of slices = 18, slice thickness = 2 mm, number of averages = 2, flip angle = 131°, bandwidth = 407 Hz/Pixel, field of view (FOV) = 160 mm^3^, scan time = 3 min and 4 s. In addition, parameters of turbo spin echo T2w images were as follows: TE/TR = 59/4000 ms, in-plane resolution = 0.6 × 0.6 mm^2^, number of slices = 18, slice thickness = 2 mm, number of averages = 2, flip angle = 160°, bandwidth = 407 Hz/Pixel, FOV = 160 mm^3^, scan time = 3 min and 6 s.

For ex vivo imaging, plaque specimens were scanned on the same 3 T scanner with a carotid surface coil. A glass container filled with 0.9% saline solution for sample preservation was used to accommodate the specimen. The protocol of CMR ex vivo imaging included T1w images, T2w images and CATCH sequences, with the following imaging parameters: (1) 2D turbo spin echo T1w images: TE/TR = 15/800 ms, in-plane resolution = 0.5 × 0.5 mm^2^, number of slices = 28, slice thickness = 1 mm, number of averages = 3, flip angle = 180°, bandwidth = 407 Hz/Pixel, FOV = 138 mm^3^, scan time = 11 min and 57 s; (2) 2D turbo spin echo T2w images: TE/TR = 46/4000 ms, in-plane resolution = 0.5 × 0.5 mm^2^, number of slices = 28, slice thickness = 1 mm, number of averages = 5, flip angle = 180°, bandwidth = 407 Hz/Pixel, FOV = 138 mm^3^, scan time = 19 min and 38 s; (3) IR prepared spoiled gradient echo sequence for CATCH: TE/TR = 5 /966 ms, in-plane resolution = 0.5 × 0.5 mm^2^, inversion time = 470 ms, number of slices = 28, slice thickness = 0.5 mm, number of averages = 1, flip angle = 12°, bandwidth = 149 Hz/Pixel, FOV = 120 mm^3^, scan time = 16 min and 31 s.

### CMR imaging analysis

Imaging quality of the in vivo images was rated on a 5-point scale according to the signal-to-noise ratio (SNR) as previously described [7]. Briefly, grade 1 was characterized as low SNR, and wall structures and the outer boundary of vessels are invisible; grade 2 was characterized as marginal SNR, and wall structures are identifiable, while outer boundaries and other substructure of arteries are indistinct; grade 3 was characterized as marginal SNR, arterial walls are visible, while outer boundaries and arterial lumen are partially obscured; grade 4 was characterized as high SNR with minimal artifacts, wall structures, arterial lumens, and outer boundaries are well identifiable; grade 5 was characterized as high SNR without apparent artifacts, arterial walls depicted well, lumens and outer boundaries are identified clearly. Images with an imaging quality grade of at least 2 were included for the study.

The definition of hemorrhage for in vivo plaque imaging was described previously [8, 9]. Briefly, the adjacent sternocleidomastoid muscle was used as the signal reference. Areas with higher signal intensity than the reference sternocleidomastoid muscle were classified as IPH, or else it was classified as non-IPH.

For ex vivo images (CATCH and T1w images), the plaque-to-saline solution ratio (PSR) value was calculated. PSR was defined as the highest signal intensity of the plaque area divided by average signal intensity of adjacent saline solution, in a manually drawn region of interest with 2 mm diameter size.

Two experienced reviewers (WY, and WL, radiologists with 10 and 3 years of experience, respectively) blinded to the histological findings assessed plaque signal intensity for both in vivo and ex vivo images. The distance between the lesion and the bifurcation was used as an anatomical reference. For the ex vivo CATCH sequence, in order to emphasize the anatomic relationship between CHIPs on gray blood CATCH sequence and bright blood CATCH sequence, gray and bright blood CATCH images were fused using a post-processing software (Horos 2.02) before the plaque signal was measured.

### Histology and microscopy

After all ex vivo CMR images were acquired, the samples were processed and sectioned as previously reported for histological analysis [7], briefly, the samples were stained with hematoxylin-eosin and Mallory’s Trichrome, with a thickness of 4 μm approximately at 2 mm interval from each specimen. The histological slides were independently evaluated by 2 reviewers who were blinded to the imaging results, to identify the existence and extent of intraplaque hemorrhage.

### CMR imaging and histology matching

To obtain accurate spatial correlation with histological slices, CMR images were divided into quadrants as previously described [7]. In vivo and ex vivo images were matched with histological results by two viewers who were experienced with both histologic slides and CMR imaging. The Receiver-operating characteristic (ROC) curve analysis was introduced for the determination of optimal PSR cutoff value matching with histological results. With reference to optimal PSR cutoff value, areas with higher PSR value on ex vivo CATCH or ex vivo T1w images were defined as CHIPs, otherwise it was defined as non-CHIPs. CHIPs on in vivo T1w images, ex vivo T1w images and ex vivo CATCH were also matched with each other. To account for the different slice thickness between in vivo T1w images (2 mm), ex vivo T1w images (1 mm), ex vivo CATCH sequence (0.5 mm) and histological sections (4 μm, every 2.0 mm), 1 histological section, 2 ex vivo T1w image sections, 4 ex vivo CATCH sections were matched with each in vivo image section, respectively. Only the locations with corresponding histology matching were used for analysis. Morphological features such as lumen shape or size, vessel wall, location or shape of calcification, and the distance to bifurcation were used as landmarks for matching MR images and histological slides.

### Statistical analysis

Continuous variables were reported as mean ± standard deviation (SD). Categorical variables were reported as counts and percentages. ROC curve analysis was introduced for the determination of optimal PSR cutoff value matching with histological results for ex vivo CATCH and ex vivo T1w images sequences. Sensitivity, specificity and Cohen’s Kappa (k) value with 95% confidence interval were analyzed to quantify the agreement detected IPH among the following CMR protocols: in vivo T1w images, ex vivo T1w images, and ex vivo CATCH, with reference to histological findings. The correlation between in vivo T1w images and ex vivo CATCH, ex vivoT1w images and ex vivo CATCH, in vivo T1w images and ex vivo T1w images was also computed. All statistical calculations were performed in SPSS (version 19.0, International Business Machines, Inc., Armonk, New York, USA) and statistical differences were significant for *p* values < 0.05.

## Results

Among 12 patients who underwent carotid endarterectomy, 11 patients successfully completed in vivo scans. Ultimately, 10 in vivo plaques and 17 ex vivo plaques were included for analysis, yielding a total of 236 in vivo locations, 328 ex vivo and matched histology locations. For in vivo samples, 86 locations contained histologically confirmed IPH (36.4%), and for ex vivo ones, 96 locations confirmed IPH (40.3%), while none of the coronary locations demonstrated IPH. The basic clinical characteristics of study patients, gross locations of IPH, and CMR imaging findings of CHIPs are represented in Table 1.

**Table 1:** Clinical characteristics, gross locations of IPH and CMR imaging findings of CHIPs

Parameter	Datum
Age(y)	61.6 ± 8.6
Male	12(70.6%)
Hyperlipidemia	14(82.4%)
Hypertension	13(76.5%)
History of CAD	11(64.7%)
History of Peripheral Artery Disease	6(35.3%)
History of Diabetes Mellitus	5(29.4%)
Current Statin User	14(82.4%)
Current Smoker	6(33.3%)
Symptom	12(70.6%)
Gross locations of IPH	
Coronary plaques	0 (0%)
Carotid plaques	96(40.3%)
PSR value	
Ex vivo CATCH	2.0 ± 1.2
Ex vivo T1w	1.5 ± 0.7

Note: values are median, mean ± SD or n (%)

*IPH* intraplaque hemorrhage, *HIP* high intensity signal, *CAD* coronary artery disease, *PSR* plaque-to-saline solution ratio

**Fig. 2:**
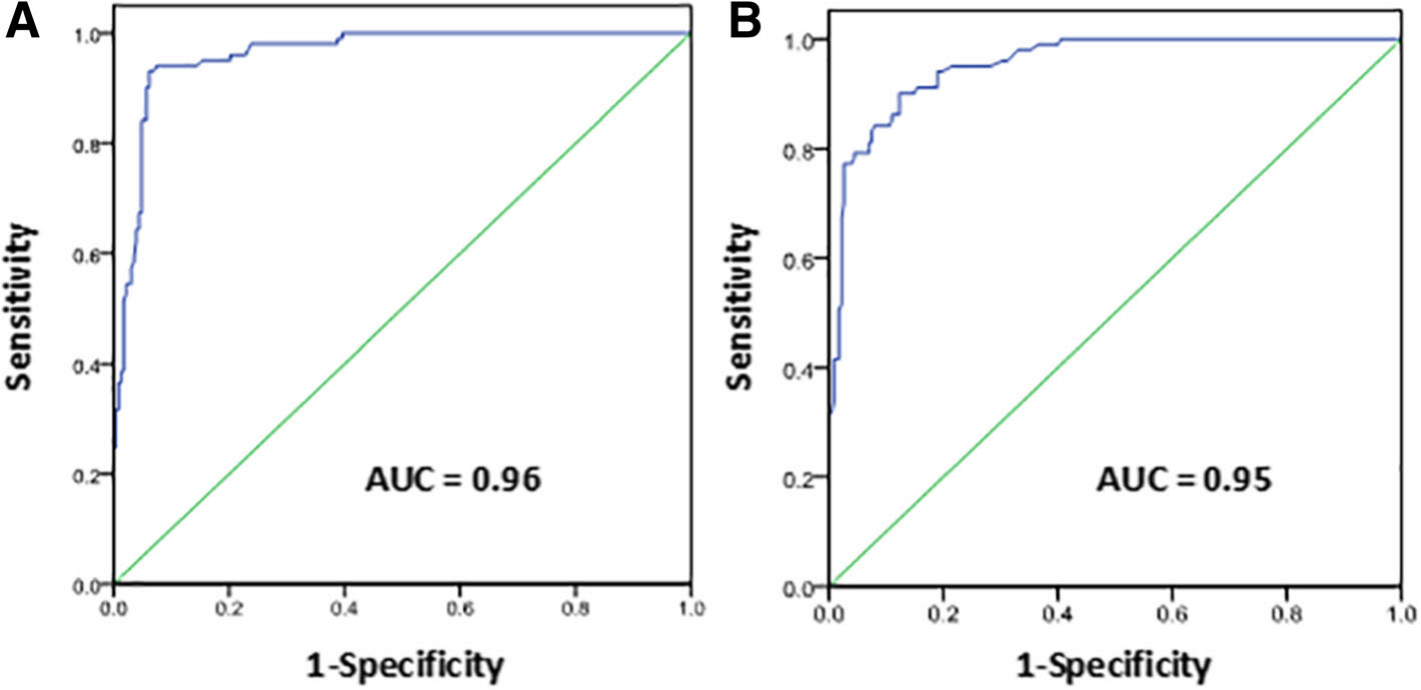
ROC curve analysis for identification of CHIP. On ROC curve analysis, the optimal cutoff value for identification of high intensity plaque on ex vivo CATCH (**a**) and ex vivo T1w images (**b**) was a PSR value of 2.0and 1.9, respectively, and the AUC was 0.96 forex vivo CATCH and 0.95 for ex vivo T1w images, yielding good sensitivity and specificity on both ex vivo CATCH and ex vivo T1w images (95.0%, 92.1% VS 77.2%, 97.4%, respectively). PSR = plaque-to-saline solution ratio value, AUC = area underneath the ROC curve

**Fig. 3:**
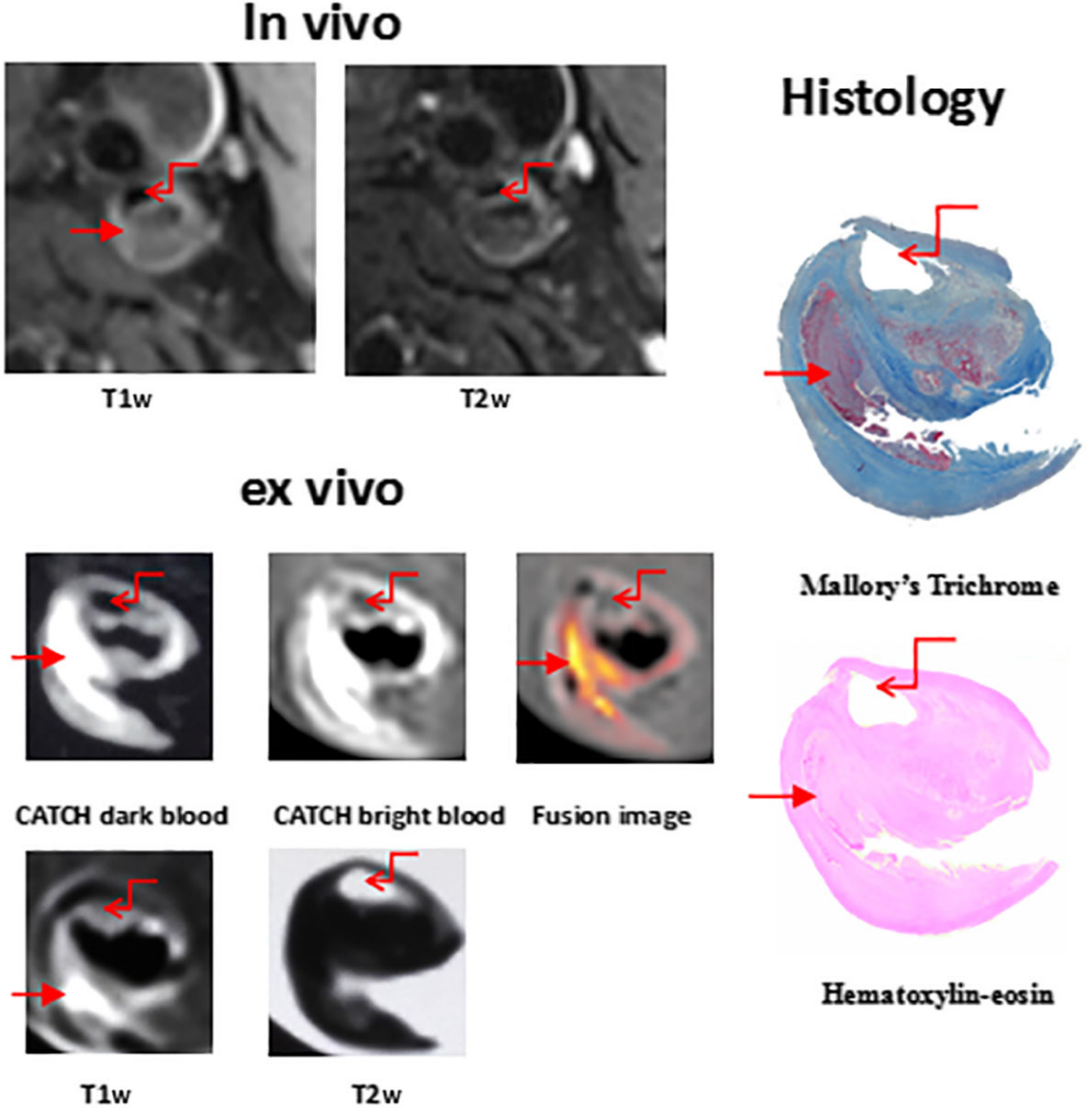
A representative case with CHIP in the internal carotid artery with  in vivo T1w images, ex vivo T1w CMR images and ex vivo CATCH (red arrow) identified in the internal carotid artery. Its corresponding histologic section with hematoxylin-eosin staining and Mallory’s trichrome staining shows IPH. Red elbow type arrow shows lumen of internal carotid artery; red arrow indicates IPH

**Fig. 4:**
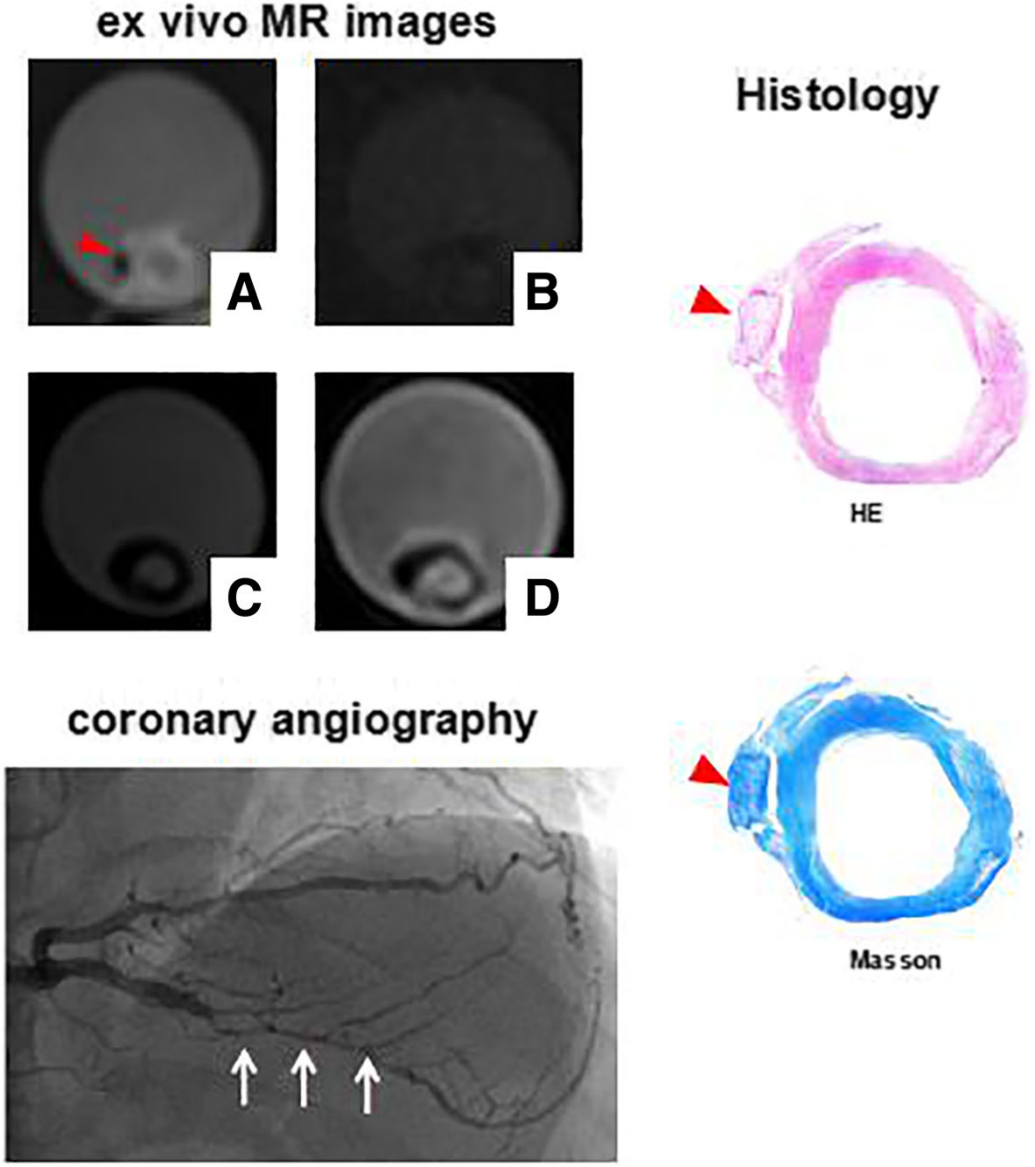
A representative case with non-CHIP in the proximal of posterior descending branch artery. In this case, x-ray coronary angiography shows severe stenosis in the proximal of posterior descending branch artery (white arrow). But none of the ex vivo CMR images demonstrates CHIP (**a**: CATCH bright blood, **b**: CATCH dark blood, **c**: T1w images, **d**: T2w images), and corresponding histologic section with hematoxylin-eosin staining and Mallory’s trichrome staining indicates non-IPH. Red arrowhead shows calcification

Compared with pathology, 66 locations demonstrated IPH for in vivo images (T1w, T2w), the sensitivity, specificity and k values were 76.7%, 95.3%, 0.75 (Tables 2, 3). According to ROC curve analysis, the optimal PSR cutoff value for identification of IPH on ex vivo CATCH and ex vivo T1w images sequences was a PSR value of 2.0 and 1.9, respectively, and the area underneath the ROC curve (AUC) was 0.96 for ex vivo CATCH and 0.95 for ex vivo T1w images (Fig. 2). With reference to this value, 78 locations demonstrated IPH for ex vivo T1w images the sensitivity, specificity and k values were 77.2%, 97.4%, 0.78, and 96 locations shows IPH for ex vivo CATCH, the sensitivity, specificity and k values were 95.0%, 92.1%, 0.84 respectively (Tables 2, 3). Moderate agreement was reached for in vivo T1w images, ex vivo T1w images and ex vivo CATCH on detection of IPH when compared with histology: in vivo T1w images and ex vivo CATCH (k = 0.68), ex vivo T1w images and ex vivo CATCH (k = 0.74), in vivo T1w images and ex vivo T1w images (k = 0.83). Figure 3 is a representative case with CHIP on both T1w images and CATCH sequences identified in the internal carotid artery and corresponding histologic section. Figure 4 is a presentative case with non-CHIP in the proximal segment of the posterior descending branch of right coronary artery.

**Table 2:** Test performance of CMR Images and corresponding histology for IPH

	Histology(+)	Histology(−)
In vivo T1w (+)	66	7
In vivo T1w (−)	20	143
Ex vivo T1w (+)	78	6
Ex vivo T1w (−)	23	221
Ex vivo CATCH (+)	96	18
Ex vivo CATCH (−)	5	209

**Table 3:** The sensitivity, specificity and k values of each CMR sequence

	Sensitivity (%)	Specificity (%)	k values
In vivo T1w	76.7	95.3	0.75
Ex vivo T1w	77.2	97.4	0.78
Ex vivo CATCH	95.0	92.1	0.84

## Discussion

This is the first study to demonstrate that CHIPs detected by CATCH on ex vivo plaques are indicative of the presence of IPH with histological validation. Our preliminary findings support the potential role of CATCH to detect IPH. Ex vivo T1w images, in vivo T1w images, and ex vivo CATCH all showed moderate agreement with histology.

### Assessment of HIPs on CATCH

In previous studies of coronary plaque imaging with T1w images, plaque to muscle ratio (PMR) value defined as the highest signal intensity of coronary plaque to adjacent left ventricular cardiac muscle was introduced as a quantitative measure of CHIPs. Areas with PMR > 1.0 were defined as CHIPs, or else they were defined as non-CHIPs [3, 12]. However, in our ex vivo CMR plaque images (CATCH and T1w images), left ventricular cardiac muscle was absent, while saline solution was used as an objective reference to quantify CHIPs. On the basis of the PMR value, the PSR, the highest signal intensities of excised plaques divided by adjacent saline solution, was calculated. And ROC curve analysis was applied to define optimal PSR cutoff value matching with histological results. In the ROC curve analysis, the optimal cutoff value for identification of IPH on both CATCH and ex vivo T1w images was PSR value of 2.0 and 1.9 respectively, and the area underneath the ROC curve (AUC) was 0.96 for ex vivo CATCH and 0.95 for ex vivo T1w images, according to ROC curve analysis (Fig. 2), yielding substantial sensitivity, specificity and agreement on both ex vivo CATCH and ex vivo T1w images while compared with histology (Table 2).

### Tissue characteristics and progress of CHIPs

CMR technique for atherosclerotic plaque imaging was originally used as a diagnostic tool for cerebrovascular disease. Many investigators have reported that CHIPs detected on T1w images in carotid artery indicates IPH containing methemoglobin, a product of hemoglobin degeneration after erythrocyte extravasation, leading to significantly T1 shortening and high signal intensity [9–11]. Furthermore, several studies have shown that CHIPs in coronary artery on T1w images are associated with the presence of mural or intraluminal hemorrhage detected by invasive coronary imaging [3, 12]. Jansen et al. reported that CHIPs detected on T1w images allowed the detection of intra-coronary thrombus correctly compared with invasive coronary angiography [12]. Matsumoto et al., using OCT as a gold standard, showed that intraluminal CHIPs on non-contrast T1w CMR images indicate thrombus and intimal vasculature, while mural CHIPs on T1w images may associate with intraplaque hemorrhage [3].

### The relationship between CHIPs on CATCH and IPH

Recently, a novel 3D whole-heart imaging tool was developed for coronary artery plaque characterization, namely CATCH, which simultaneously obtained heavily grey-blood T1w images along with bright-blood reference images in an interleaved fashion. The preliminary comparison with OCT indicated the positive association between CHIPs on CATCH and vulnerable plaque features such as large lipid pools and macrophage accumulation. Unfortunately, the patient population enrolled in this study all presented with stable angina with none of the subjects presenting with intracoronary thrombus detected on OCT [5]. Therefore it was unclear from that study whether the presence of CHIPs on CATCH indicates IPH with histopathologic validation.

Our study demonstrated that CHIPs detected on CATCH for ex vivo plaques are highly indicative of the presence of IPH, yielding good sensitivity, specificity and agreement compared with histology (Table 2). Moderate agreement was also reached between ex vivo CATCH and in vivo T1w images (κ = 0.68), and ex vivo T1w images (κ = 0.74), respectively. The CATCH technique features an interleave acquisition scheme with dark-blood T1w images and bright-blood T2w images in an approximately 10-min scan. Dark-blood T1w images is used for the identification of IPH, while bright-blood T2w images is used as anatomical reference [5]. Our study demonstrates IPH indicated CHIPs on dark-blood T1w images, which was consistent with previous conventional T1w images sequence [3, 12]. Furthermore, this study also shows a slightly higher sensitivity, specificity and k value with ex vivo CATCH than in vivo T1w images and ex vivo T1w images with statistical significance. Such improvement may result from the stronger T1-weighting from CATCH to heavily suppress blood, fat, and background tissues. Moreover, signal of plaque components such as lipid-rich necrotic core and fibrous tissue can also be suppressed by CATCH sequence, owing to their relatively long T1 characteristics [13, 14].

Unfortunately, none of the coronary plaque locations in this study show IPH. This may be because the coronary plaque specimens were gained from patients who underwent coronary artery endarterectomy suffered from mural extensive and diffuse coronary artery disease [15]. Fuster et al. described the pathologic process of this type of coronary plaques as phase 5 (types Vb and Vc lesions), characteristic as severely stenotic or occlusive fibrotic lesions [16] which presented as non-HIP on T1w images and CATCH.

### Limitations

In this preliminary study to evaluate CATCH sequence for IPH detection, several limitations can be identified prompting further study and validation. First, the sample size of plaque specimen is relatively small, which resulted in a limited statistical power requiring further validation via a much larger sample size. Though our sample size was limited, CATCH was able to demonstrate moderate agreement with histology with a relatively high sensitivity, specificity, and Cohen’s K. Second, in the previous study of coronary CMR plaque imaging, left ventricular cardiac muscle was used as objective reference and PMR value was introduced as a quantitative manner of CHIPs [3, 12]. This is in comparison with our ex vivo images (CATCH and T1w images), in which the left ventricular cardiac muscle was absent. Saline solution was used instead as an objective reference to quantify CHIPs. Therefore, the threshold for CHIPs was different from previous study and the results should be interpreted cautiously. Third, all specimen slices with IPH were identified from carotid plaques, while none of coronary plaques indicated CHIPs. Although the negative predictive value was validated from coronary plaques, the positive predictive value could only have verified from carotid plaques, which is indirect evidence due to potentially different pathophysiological course between carotid and coronary vessels. Therefore, additional validation studies from specifically coronary plaques are required. Fourth, the ROC analysis was performed in the same sample and a further prospective validation was needed in another sample. Furthermore, our study uses saline solution as nutrient media for sample preservation, which is different from in vivo condition and may result in components change of atherosclerotic plaques. Lastly, the ex vivo images (CATCH and T1w images) would have been limited by the same fluid being in the lumen and surrounding tissue unlike in vivo where there would be blood and tissue. In addition, T1 relaxing time of saline solution might differ from in vivo conditions and they all could influence the identification of CHIPs on ex vivo CATCH and T1w images. Furthermore, compared with in vivo conditions, the surgical excision process and tissue preparation course may lead to tissue distortion.

## Conclusions

This preliminary study demonstrates that CHIPs on CATCH in ex vivo carotid plaques are highly indicative of the presence of IPH with excellent accuracy as evidenced by histopathological validation. The results support CATCH’s potential role in detecting IPH.

## Abbreviations

3D: Three dimensional
ACS: Acute coronary syndrome
AUC: The area underneath the ROC curve
CABG: Coronary artery bypass graft surgery
CATCH: Coronary atherosclerosis T1-weighted characterization with integrated anatomical reference
CHIP: Coronary high intensity plaque
CMR: Cardiovascular magnetic resonance
FOV: Field of view
IPH: Intraplaque hemorrhage
IR: Inversion recovery
PMR: plaque to muscle ratio
PSR: Plaque-to-saline solution ratio
ROC: Receiver-operating characteristic
SD: Standard deviation
SNR: Signal-to-noise ratio
T1w: T1-weighted
T2w: T2 weighted
TE: Echo time
TR: Repetition time
